# Nano-sized carriers in gene therapy for peritoneal fibrosis *in vivo*

**DOI:** 10.1080/20022727.2017.1331100

**Published:** 2017-06-15

**Authors:** Yusuke Igarashi, Taro Hoshino, Susumu Ookawara, Kenichi Ishibashi, Yoshiyuki Morishita

**Affiliations:** a Division of Nephrology, Department of Internal Medicine, Jichi Medical University, Tochigi, Japan; b Division of Nephrology, First Department of Integrated Medicine, Saitama Medical Center, Jichi Medical University, Saitama, Japan; c Department of Medical Physiology, Meiji Pharmaceutical University, Tokyo, Japan

**Keywords:** Peritoneal fibrosis, gene therapy, viral vector, non-viral vector

## Abstract

Peritoneal fibrosis is a crucial complication in patients receiving peritoneal dialysis. It is a major pathological feature of peritoneal membrane failure, which leads to withdrawal of peritoneal dialysis. No specific therapy has yet been established for the treatment of peritoneal fibrosis. However, gene therapy may be a viable option, and various nano-sized carriers, including viral and non-viral vectors, have been shown to enhance the delivery and efficacy of gene therapy for peritoneal fibrosis *in vivo*. This review focuses on the use of nano-sized carriers in gene therapy of peritoneal fibrosis *in vivo*.

## 1. Introduction

Peritoneal dialysis is a home-based renal replacement therapy for patients with end-stage renal disease [1]. The procedure involves injecting a peritoneal dialysis solution into the abdominal cavity through an inserted peritoneal dialysis catheter and using the peritoneum as a dialysis membrane for ultrafiltration and solute clearance [1]. Peritoneal dialysis has certain advantages over hemodialysis, such as convenience, economy, and a shorter dialysis time [1,2]. However, peritoneal membrane failure represents a major obstacle to continued long-term peritoneal dialysis [3–6]. Peritoneal membrane failure manifests as deleterious structural and functional alterations caused by exposure to bio-incompatible peritoneal dialysis solutions [3–6]. Peritoneal fibrosis is a major pathological feature of peritoneal membrane failure [6–8], characterized histologically by myofibroblast proliferation and excess accumulation of extracellular matrix, including collagen, in the peritoneal mesothelium [9,10]. No specific therapy has yet been established for the treatment of peritoneal fibrosis. However, numerous cell types, including mesothelial cells, bone marrow-derived cells, endothelial cells, and fibroblasts, have been reported to contribute to its development [11], and *in vivo* studies aimed at improving our understanding of the potential therapeutic approaches for peritoneal fibrosis are urgently required. Gene therapy may be a potential therapeutic option because it can target novel molecules that were previously difficult to target using small molecules or antibodies. Various nano-sized carriers, including viral and non-viral vectors, have been shown to enhance the delivery and treatment effects of gene therapy [12,13]. This review focuses on the use of nano-sized carriers in gene therapy of peritoneal fibrosis *in vivo*.

## 2. Mechanism of peritoneal fibrosis development

**Figure 1.**
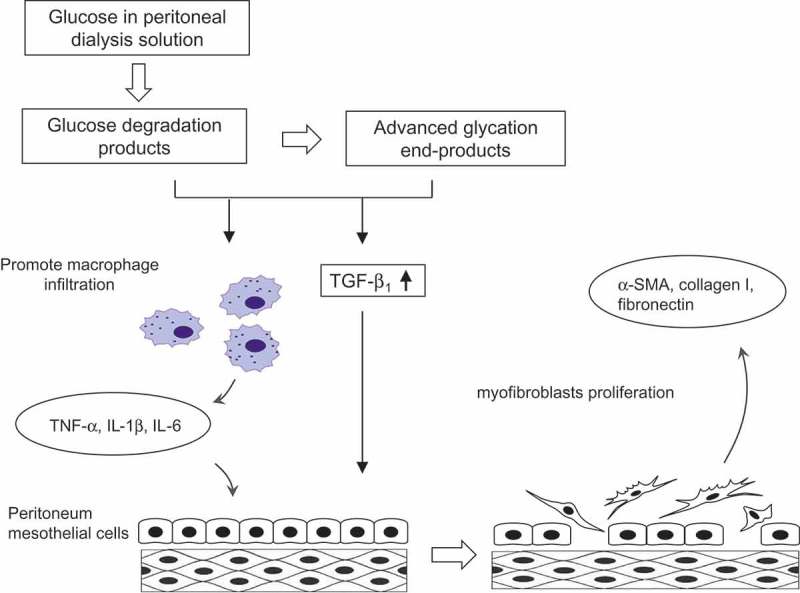
Mechanism of peritoneal fibrosis development. TGF: transforming growth factor; TNF: tumor necrosis factor; IL: interleukin; SMA: smooth muscle actin.

The mechanism of peritoneal fibrosis is shown in Figure 1. Repeated exposure to peritoneal dialysis solutions containing high concentrations of glucose is considered to play a central role in the development of peritoneal fibrosis in patients undergoing peritoneal dialysis [3–6]. Glucose is degraded to glucose-degradation products including methylglyoxal, glyoxal, formaldehyde, and 3-deoxyglucosone during heat sterilization [14–16], and these products are further transformed to advanced glycation end-products [14,15,17–19]. Both the glucose-degradation products and advanced glycation end-products have been reported to activate transforming growth factor (TGF)-β_1_ signaling in the peritoneal membrane, thus promoting peritoneal fibrosis [14,15,17–20]. Activated TGF-β_1_ promotes the proliferation of fibroblasts from different origins, including mesothelial cells via mesothelial–mesenchymal transition, bone marrow-derived cells, and endothelial cells, in addition to resident fibroblasts [21–24]. TGF-β_1_ also increases the production of various extracellular matrix and fibrogenesis-associated molecules such as Snail, fibronectin, collagen I, and α-smooth muscle actin (α-SMA) on the peritoneal membrane, leading to peritoneal fibrosis [21–24]. Both glucose-degradation products and advanced glycation end-products have also been reported to promote chronic inflammation characterized by infiltration of macrophages [25,26], which in turn secrete pro-fibrotic cytokines such as tumor necrosis factor-α (TNF-α), interleukin (IL)-1β, and IL-6, as well as TGF-β_1_ [27–31]. These cytokines induce peritoneal fibrosis by promoting fibroblast proliferation and type I collagen synthesis on the peritoneum [32,33].

## 3. Nano-sized carriers for gene therapy of peritoneal fibrosis *in vivo*

**Figure 2.**
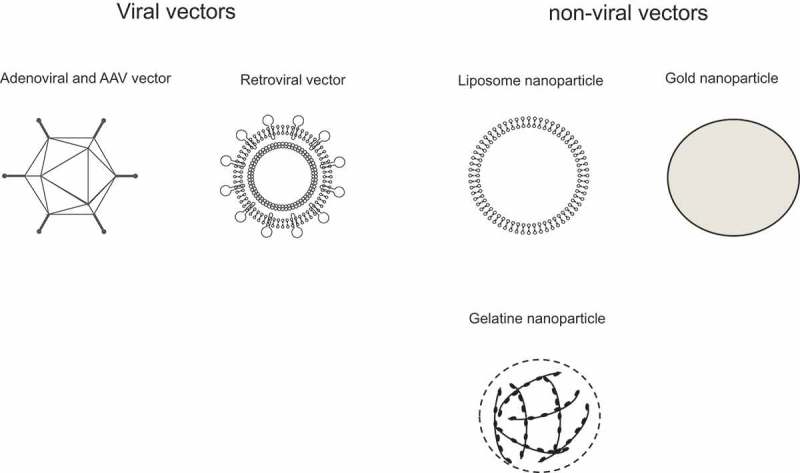
Viral and non-viral vectors for renal fibrosis *in vivo*.

Various nano-sized carriers, including viral and non-viral vectors, have been studied for the gene therapy of peritoneal fibrosis (Figure 2) [34–45]. The different categories of vectors, transgenes, and administration routes, and their effects on peritoneal fibrosis *in vivo* are summarized in Table 1.

**Table 1.** Gene therapies and nano-sized carriers for the treatment of peritoneal fibrosis.

Vector		Transgene	Administration route	Effects	Authors (published year)	Reference No.
Virus vectors	Adenovirus	Angiostatin-DNA	Intraperitoneal injection	Ameliorated peritoneal fibrosis by inhibiting angiogenesis	Hoff and Margetts (2006)	[34]
	Adenovirus	Angiostatin-DNA	Intraperitoneal injection	Ameliorated peritoneal fibrosis by inhibiting angiogenesis	Margetts et al. (2002)	[35]
	Adenovirus	Decorin-DNA	Intraperitoneal injection	Inhibited collagen accumulation in peritoneum without improving ultrafiltration ability	Margetts et al. (2002)	[35]
	Adenovirus	BMP-7-DNA	Intraperitoneal injection	Ameliorated peritoneal fibrosis	Yu et al. (2009)	[36]
	AAV	Decorin-DNA	Intraperitoneal injection	Inhibited peritoneal fibrosis with preservation of peritoneal cell size, decreased peritoneal thickness, and decreased expression of α-SMA	Chaudhary et al. (2014)	[37]
	Retrovirus	CTGF-siRNA	Applied to cultured human peritoneal mesothelial cells	Inhibited production of the extracellular matrix such as fibronectin, collagen I and laminin and VEGF expression under stimulation of TGF-β_1_	Xiao et al. (2010)	[38]
Non-viral vectors	Liposome nanoparticles	TGF-β_1_-siRNA	Intraperitoneal injection	Inhibited TGF-β_1_ expression in peritoneum, and inhibited peritoneal fibrosis with decreased proliferation of α-SMA-positive myofibroblasts	Yoshizawa et al. (2015)	[39]
	Gold nanoparticles	Decorin-DNA	Intraperitoneal injection	Inhibited peritoneal fibrosis	Chaudhary et al. (2014)	[37]
	Cationic gelatin nanoparticles	HSP47-siRNA	Intraperitoneal injection	Inhibited expression of HSP47 on peritoneum, and inhibited peritoneal fibrosis with decreased expression of type III collagen, TGF-β_1_, α-SMA, and MCP-1	Obata et al. (2012)	[40]
Naked, artificial, modified oligonucleotides	None	HSP47-antisense oligonucleotides	Intraperitoneal injection	Inhibited expression of HSP47, and inhibited peritoneal fibrosis with decreased expression of types Ⅰ and Ⅲ collagen and α-SMA, and inhibited macrophage infiltration	Nishino et al. (2003)	[41]
	None	MicroRNA-21-5p-inhibitor-BNA	Intraperitoneal injection	Inhibited peritoneal fibrosis with increased expression of PPAR-α	Morishita et al. (2016)	[42]
Ultrasound-microbubble-mediated transfer	None	Smad7-plasmid DNA	Intraperitoneal injection	Inhibited peritoneal fibrosis	Guo et al. (2007)	[43]
	None	MicroRNA-30a	Intraperitoneal injection	Inhibited peritoneal fibrosis with inhibition of Snail signaling pathway	Zhou et al. (2013)	[44]
	None	MicroRNA-29b	Intraperitoneal injection	Inhibited peritoneal fibrosis with inhibition of TGF-β_1_ signaling pathway	Yu et al. (2014)	[45]

BMP-7: bone morphogenic protein-7; AAV: adeno-associated viral vector; α-SMA: α-smooth muscle actin; CTGF: connective tissue growth factor; VEGF: vascular endothelial growth factor; IκB-α: I-kappa-B-alpha; TGF-β_1_: transforming growth factor-β_1_; HSP47: heat shock protein 47; MCP-1: monocyte chemoattractant protein-1; PPAR-α: peroxisome proliferator-activated receptor; Smad7; mothers against decapentaplegic homolog 7.

### 3.1. Viral vectors

Various viral vectors have been investigated for peritoneal fibrosis, including adenoviral, adeno-associated viral (AAVs), and retroviral vectors.

### 3.2. Adenoviral vectors

Adenoviral vectors are one of the most widely studied viral vectors for gene therapy of peritoneal fibrosis *in vivo*. Adenoviral vectors are double-stranded, non-enveloped DNA viral vectors of 70–90 nm in diameter, with a genome of 36–38 kb [46,47]. Transgenes can be inserted into the DNA sequence of adenoviral vectors [46,47], which are then transfected into cells via receptor-mediated endocytosis [46,47]. Adenoviral vectors have many advantages in terms of gene delivery, including high transduction efficiency and a large capacity for transgene insertion into their DNA. However, the high expression efficiency of transgenes delivered using adenoviral vectors is transient because the transgenes are not integrated into the host genome by these vectors [46,47]. However, adenoviral vectors have been reported to deliver transgenes to the peritoneal membrane with high efficiency in peritoneal fibrosis rodent models, and intraperitoneal administration of adenoviral vectors expressing the angiogenesis inhibitor, angiostatin, inhibited peritoneal fibrosis by inhibiting angiogenesis in rodent models [34,35]. Another study reported that intraperitoneal administration of adenoviral vectors expressing decorin, which blocks TGF-β_1_ signaling, inhibited collagen accumulation in the peritoneum but failed to improve the ultrafiltration rate of the peritoneal membrane in a rat peritoneal fibrosis model [35]. Intraperitoneal administration of adenoviral vectors expressing bone morphogenetic protein-7 (BMP-7), which is an anti-fibrotic molecule, was also shown to ameliorate peritoneal fibrosis in a rat model [36]. In that study, adenoviral vector-mediated BMP-7 delivery maintained increased expression levels of BMP-7 in the peritoneum for up to 14 days after administration [36], and inhibited mesothelial–mesenchymal transition of cultured human peritoneal mesothelial cells [36].

### 3.3. Adeno-associated viral vectors

AAVs are single-stranded, non-enveloped DNA viral vectors 18–26 nm in diameter, with a genome of 4–5 kb [46,48,49]. AAVs can deliver transgenes into both dividing and non-dividing cells, and can incorporate their transgenes into the host genome [46,48,49]. Intraperitoneal administration of AAVs expressing decorin significantly inhibited peritoneal fibrosis, associated with preserved peritoneal cell size, decreased peritoneal thickness, and decreased expression of α-SMA in the peritoneum in a mouse peritoneal fibrosis model [37].

### 3.4. Retroviral vectors

Retroviral vectors are enveloped RNA viral vectors 80–130 nm in diameter, with a genome of 8–11 kb [50]. Retroviral vectors can deliver transgenes into the cell via an interaction between their envelope and cell surface receptors [50]. Unlike adenoviral vectors, retroviral vectors only deliver transgenes into dividing cells [50]. They can incorporate transgenes into the host genome and are therefore capable of long-term transgene expression [38,50]. Cultured human peritoneal mesothelial cells transfected with small interfering RNA (siRNA) targeted to connective tissue growth factor (CTGF) using retroviral vectors knocked down CTGF expression and inhibited extracellular matrix production, including fibronectin, collagen I, and laminin, as well as vascular endothelial growth factor expression under stimulation by TGF-β_1_ [38]. These results suggest that retroviral vector-mediated CTGF knockdown in peritoneal mesothelial cells may be a promising tool for preventing peritoneal fibrosis *in vivo*. However, the therapeutic effects of retroviral vector-mediated transgene delivery on peritoneal fibrosis have not been investigated *in vivo*.

## 4. Non-viral vectors

Several studies have reported on the possible effects of gene therapies using non-viral vectors for the treatment of peritoneal fibrosis. Non-viral vectors have the advantages of less immunogenicity and toxicity than viral vectors when administered *in vivo*. In addition, their preparation is relatively simple compared with viral vectors. Non-viral vectors used for the treatment of peritoneal fibrosis *in vivo* include liposome nanoparticles [39], gold nanoparticles [37], and cationic gelatin nanoparticles [40], which have been shown to deliver transgenes to the peritoneum effectively in peritoneal fibrosis animal models and have demonstrated efficacy *in vivo* (Figure 2) [37,39,40].

### 4.1. Liposome nanoparticles

Liposome nanoparticles consist of phospholipids and cholesterol, which are the main components of the cell membrane, and thus show high biocompatibility [39,51,52]. Liposome nanoparticles have been reported to deliver transgenes to the peritoneal membrane, and demonstrated therapeutic efficacy in a mouse peritoneal fibrosis model [39]. Intraperitoneal administration of liposome nanoparticles encapsulating TGF-β_1_-siRNA knocked down TGF-β_1_ expression in the peritoneum and inhibited peritoneal fibrosis, associated with decreased proliferation of α-SMA-positive myofibroblasts derived from different cell types, including mesothelial and bone marrow-derived cells [39].

### 4.2. Gold nanoparticles

Gold nanoparticles comprise a colloidal gold suspension in a fluid and have demonstrated high stability, low toxicity, and low immunogenicity [53]. They were shown to deliver transgenes to the peritoneum for the treatment of peritoneal fibrosis *in vivo* [37]. Intraperitoneal administration of plasmid DNA expressing decorin with gold nanoparticles inhibited peritoneal fibrosis by inhibiting the effects of TGF-β_1_ in a rat peritoneal fibrosis model [37].

### 4.3. Cationic gelatin nanoparticles

Gelatin is a protein derived from collagen [54]. Cationic gelatin nanoparticles are produced chemically by introducing cations such as ethylenediamine, putrescine, spermidine, or spermine to the carboxyl group of gelatin, and have been shown to protect transgenes against degradation *in vivo* [40]. The release rate of transgenes from cationic gelatin nanoparticles can be modulated by changing the degradability of the gelatins [40]. Cationic gelatin nanoparticles demonstrated therapeutic efficacy in a mouse peritoneal fibrosis model [40]. Intraperitoneal single injection of heat shock protein 47 (HSP47)-siRNA entrapped with cationic gelatin nanoparticles was shown to release HSP47-siRNA continuously over 21 days as a result of degradation of the gelatin nanoparticles [40]. They also significantly inhibited both expression of HSP47 in the peritoneum and peritoneal fibrosis, together with decreased expression of type III collagen, TGF-β_1_, α-SMA, and monocyte chemoattractant protein-1 in peritoneal tissue in a mouse peritoneal fibrosis model [40].

## 5. Other methods of gene therapy for renal fibrosis *in vivo*

Intraperitoneal injection of naked, artificial, modified oligonucleotides has shown therapeutic effects against peritoneal fibrosis *in vivo* [41,42]. Ultrasound-mediated transgene delivery also resulted in effective delivery of transgenes to the peritoneum and therapeutic effects against peritoneal fibrosis *in vivo* [43].

### 5.1. Naked artificial modified oligonucleotides

Intraperitoneal injection of naked, antisense oligonucleotides and artificially synthesized bridged nucleic acids (BNA) inhibited peritoneal fibrosis *in vivo* [41,42]. Antisense oligonucleotides are short, artificial synthetic 15–25 nt oligonucleotides [55], which include phosphorothioate linkages that confer nuclease resistance to enhance intracellular stability [55]. BNAs are modified RNA nucleotides including a molecule with a five- or six-membered bridged structure with a fixed C3'-endo sugar puckering [56]. BNAs increase the binding affinities to target oligonucleotides and transgene stability [56]. Intraperitoneal injection of naked HSP47 antisense oligonucleotides inhibited peritoneal fibrosis in a rat model, associated with reduced expression levels of HSP47, types I and III collagen, and α-SMA, as well as reducing the number of infiltrating macrophages in a rat peritoneal fibrosis model [41]. In that study, HSP47 antisense oligonucleotides were shown to inhibit HSP47 expression in cells, including fibroblasts, in the peritoneal sub-mesothelial zone [41]. Intraperitoneal injection of naked, artificially synthesized BNAs of microRNA (miRNA)-21 inhibitor inhibited peritoneal fibrosis by inhibiting proliferation of myofibroblasts from different origins, such as mesothelial cells, bone marrow-derived cells, and endothelial cells, in addition to resident fibroblasts, and increased expression of the miRNA-21 target gene, peroxisome proliferator-activated receptor, in a peritoneal fibrosis mouse model [42].

### 5.2. Ultrasound-microbubble-mediated transfer

Ultrasound-microbubble-mediated gene transfer has been reported to deliver transgenes to the peritoneum and to have therapeutic effects against peritoneal fibrosis *in vivo* [43]. Plasmid DNA expressing mothers against decapentaplegic homolog 7 (Smad7) mixed with albumin-stabilized perfluorocarbon gas microbubbles was injected intraperitoneally, and the surface of the abdomen was then exposed to ultrasound from the costal margin to the pubic symphysis, resulting in overexpression of Smad7 in the peritoneum, adipose tissue, mesentery, greater omentum, and spleen, but not in the liver, kidney, pancreas, or intestine in a rat model [43]. This delivery method was shown to maintain overexpression of Smad7 in the peritoneum for up to 2 weeks and inhibited peritoneal fibrosis in a rat peritoneal fibrosis model [43]. Ultrasound-microbubble-mediated delivery of plasmid DNA expressing miRNA-29b and miRNA-30a to the peritoneum induced overexpression of the respective miRNAs in the peritoneum and inhibited mesothelial–mesenchymal transition of peritoneal mesothelial cells, resulting in inhibition of peritoneal fibrosis in a mouse peritoneal fibrosis model, by inhibiting TGF-β_1_ and Snail signaling pathways, respectively [44,45].

## 6. Summary

Various delivery systems for gene therapy of peritoneal fibrosis have been developed. However, their long-term efficacy, effects on other organs, and possible adverse effects remain unclear. In addition, no study has yet reported on vectors that can specifically target the peritoneum. Further studies are therefore needed to investigate these aspects and to develop delivery systems suitable for delivering transgenes exclusively to the peritoneum.
